# Laser-Based QR Code Marking on Double Film-Coated Tablets: Balancing Marking Efficiency and Tablet Integrity—A Step Toward Safer Medicines

**DOI:** 10.3390/pharmaceutics18010073

**Published:** 2026-01-06

**Authors:** Hadi Shammout, Béla Hopp, Tamás Smausz, János Bohus, Orsolya Jójárt-Laczkovich, Martin Cseh, Judit Kopniczky, Balázs Tari, Ranim Saker, Katalin Kristó, Tamás Sovány, Krisztina Ludasi

**Affiliations:** 1 Institute of Pharmaceutical Technology and Regulatory Affairs, University of Szeged, Eötvös u. 6, H-6720 Szeged, Hungary; hadishammout1@gmail.com (H.S.); jojartne.laczkovich.orsolya@szte.hu (O.J.-L.); cseh.martin@szte.hu (M.C.); rnmsaker@gmail.com (R.S.); kristo.katalin@szte.hu (K.K.); ludasi.krisztina@szte.hu (K.L.); 2 Department of Optics and Quantum Electronics, University of Szeged, Dóm tér 9, H-6720 Szeged, Hungary; b.hopp@physx.u-szeged.hu (B.H.); tomi@physx.u-szeged.hu (T.S.); jkopniczky@titan.physx.u-szeged.hu (J.K.); 3 ELI ALPS, The Extreme Light Infrastructure ERIC, Wolfgang Sandner u. 3, H-6728 Szeged, Hungary; janos.bohus@eli-alps.hu (J.B.); tari.balazs@eli-alps.hu (B.T.)

**Keywords:** laser technology, pharmaceutical industry, marking of SDFs, ablation, QR code, enteric film coating, ultra femtosecond laser, anti-counterfeiting

## Abstract

**Background/Objectives**: Laser has a prominent place in pharmaceutical industry, especially in the marking of solid dosage forms (SDFs). To combat falsified medicines, this study evaluates QR code marking on the surface of tablets as a supplement to serialization on packaging, using an ultrafast laser to achieve industrially relevant marking speeds while preserving the functional integrity of double film-coated ibuprofen tablets. **Methods**: Tablets were directly compressed and coated with a double film: the inner layer was a gastro-resistant coating (Acryl-EZE^®^ MP), while the outer one was a coloured, TiO_2_-containing (TC) or TiO_2_-free (TF) immediate-release coating (Opadry^®^). QR codes were ablated on the tablet surface using various laser parameters (e.g., pulse energy and scanning speed), and the effects were physically, chemically, and microscopically examined to evaluate their properties after this processing. **Results**: No significant differences were observed between TC and TF coatings. In addition, the readability of QR code is strongly influenced by laser settings and coating types. Furthermore, the used laser has achieved the expected fast marking speed and high-precision coding, which may be economically feasible for pharmaceutical companies. According to the profilometry findings, the ablation depth could be compensated for with an appropriate coating thickness to enable the desired release properties. This was confirmed by the results of SEM, Raman analysis, and in vitro dissolution test. **Conclusions**: Ultrafast Ti:Sa laser-based QR code marking directly onto the dosage form offers increasing benefits in the healthcare field. However, it may undesirably affect the behavior of the dosage form. This requires careful consideration of formulation and laser processing conditions before application, especially in the case of delayed-release (DR) systems.

## 1. Introduction

Laser technology made the achievement of several previously unachievable tasks possible. Its unique characteristics, including monochromaticity, collimation, and coherence, enabled its widespread use in a wide range of applications, from the smallest lasers in a compact disc player to the largest lasers utilized in various industries [1,2,3,4].

Laser technology has penetrated various fields of pharmaceutics. Applications range from sterilization in the deep ultraviolet range [5,6,7] and cleaning-process validation [8] to analytical techniques [9], active-molecule discovery [10,11], three-dimensional two-photon polymerization (TPP) for micro/nanoscale devices [12,13], and packaging technologies [14,15]. Recently, a novel laser cutting technology has been used for on demand production of oral solid dosage forms (OSDFs), [16], osmosis-controlled release systems [9,17], and even solid-state modification of active pharmaceutical ingredients (APIs) [18,19,20,21]. Laser applications extend even further, playing a key role in particle size reduction [22,23], drug delivery [24,25], and numerous 3DP technologies [26].

In addition, laser markings or coding technologies, which application are the focus of the current research paper, are also emerging in the pharmaceutical industry. Laser marking is a noncontact process that uses a focused beam of high-powered laser to create permanent and readable (numeric, alphabetic, alphanumeric, etc.) markings on the surface of different materials by altering their appearance or properties. This process can be applied to a wide range of materials (plastics, metal, glass, ceramics, etc.) [27,28,29] and offers many advantages over traditional embossing and printing techniques [30]. Among the most significant advantages are the range of features that can be placed on the surface [31,32] and the elimination of the need for organic solvents [33]. In addition, it is not affected by the environmental conditions of the processing room and the requirements of the ink design (viscosity, temperature, surface tension, etc.), which can make the process more reproducible [34]. The absence of direct contact between the laser and the material minimizes contamination and defects. [31,33,34]. The resulting marks are also clearer, more durable, and resistant to heat or chemicals [28,29]. Further benefits include no ink-related costs and the elimination of concerns about the use of unsafe or non-pharmaceutically approved materials [33,34]. Moreover, it can produce extremely fine details and provides excellent potential for automation in high-speed applications [29,35]. Therefore, laser marking is considered the current choice for the pharmaceutical industry.

However, not all lasers are equally suited for marking. For instance, infrared (IR) lasers are not suitable for heat-sensitive materials because of the intense localized heat they produce during application. Additionally, both of pulsed neodymium: yttrium-aluminum-garnet laser (Nd:YAG) and semiconductor lasers have been found to cause significant damage (burning) of the coating structure. Although excimer UV lasers are free from all of the above disadvantages, they require expensive maintenance and equipment costs [33,34].

To mark any material, at least part of the laser beam must be absorbed directly by or near the surface [30]. The interaction between the surface of a solid object and the laser beam depends on wavelength, the power of the laser, exposure time, and optical properties of the marked object [30,36]. Several mechanisms can be distinguished when marking a material, like ablation, melting, burning, discoloration, or a combination of some or all of them. These mechanisms induce various structural changes in materials [30,37]. This study utilizes laser ablation to mark SDFs, which is a top-down process of removing material by focusing a laser beam onto a substrate. Cozzens was the first to publish a report on the laser ablation of polymers in 1977, where he used an IR laser source (λ = 10.6 μm) to apply it to 11 polymers [38]. Subsequently, research studies have started to explore this mechanism and its applications in greater depth. Ablation is a clean and cost-effective method suitable for TC and TF coatings. The ablation itself results in low marking contrast, as there is no color change in the treated area, so it is necessary to coat the tablet with at least one differently colored layer as the tablet. By selective removal of this layer using laser, the marking become visible [30].

Laser marking technology also plays a key role in personalized medicine by placing patient-specific marks or information tailored to each patient on the surface of the dosage form. This is important when dose flexibility is required, based on weight, gender, age, or genetic factors, and it can enhance medication safety, especially for elderly patients managing multiple medications [33,39]. This technology is also useful for brand recognition, reducing the incidence of medical errors [40,41]. Marking/coding can have other applications, such as tracking medications across the distribution chain or remote monitoring [42]. Furthermore, one of the most interesting applications of laser marking in the pharmaceutical industry is the combating of drug counterfeiting [33,34,43].

Counterfeiting is a global problem whose magnitude is difficult to quantify. It poses a real threat to health, economies, and society. Unfortunately, this phenomenon appears to be gradually worsening in countries of the European Union (EU). Consequently, compliance with the requirements of the Commission Delegated Regulation (EU) 2016/161 and Directive 2011/62/EU of June 2011 has become mandatory since 9 February 2019 [44,45]. The goal is to apply serialization and verification in order to reduce the scale of this problem [33,34,43] by placing unique identifiers on each medicine package for traceability purposes. Moving forward, a possible objective is to enable the placing of these codes directly on the surface of dosage forms, so the patient can scan them using a mobile phone for additional verification and authentication of the medicine, even in the absence of original packaging [33,34]. In this situation, tablet-level QR marking by laser is considered as a complement to legally mandated pack-level serialization, particularly for small, high-value batches and hospital/clinical personalization, rather than a replacement. This procedure also avoids the problem of repackaging or replacing the package or blister card illegally [46,47]. However, this code should not affect the performance of the dosage form during marking [34].

Several research studies have indicated that marking process can be applied directly to dosage forms [39,47,48,49,50], and previous studies of our research group also support this [33,34,43]. Our research group has successfully marked quick response codes (QR codes) on the surface of enteric-coated tablets using several types of lasers, which is currently one of the most popular methods of tracking medicines and patients in healthcare, mainly to improve patient safety [39,46,48,51]. This type of code can contain information in both directions (vertical and horizontal) and provides all-around/360-degree reading [43,47]. In addition, it can store a large amount of information (with the ability to update it) in a small, 2D space with high reliability, which can be easily accessed using a special scanner [48,52]. In the case of tablets, the name of the API, indication, dosage, batch number, expiration date, possible interactions, physician instructions, method of storage, or even specific information for each patient, can be encoded [39,41,43,48,49]. They do not require fluorescent materials in the formulation, thus simplifying the manufacturing process and also having a positive impact on costs [47]. They also facilitate medical screening and the ability to access information at the point of care or on demand using smartphones [39,46,47,49]. Moreover, this type of code was proposed to prevent drug counterfeiting [39,43,46,49]. Recently, QR code was implemented in the electronic delivery of patient information leaflets [52].

**Figure 1:**
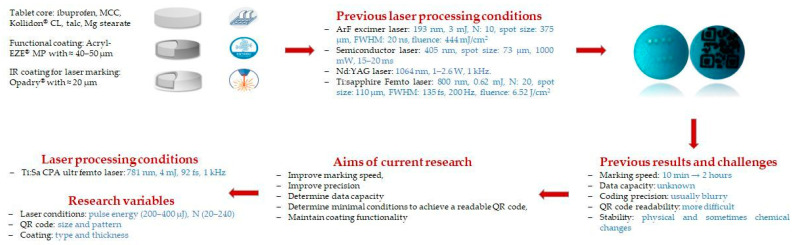
The visual schematic of the double-coating architecture, laser ablation strategy, and previous and current challenges.

However, it was pointed out that for enteric-coated tablets, an additional coating layer of a different color was required to achieve successful marking, without the corruption of coating functionality. Furthermore, the marking speed was relatively low for most of the tested lasers, and the presence of TiO_2_ in the coatings caused reading problems since, for certain types of lasers, the energy could not be raised above the ablation threshold of the TiO_2_. Therefore, selecting the appropriate laser type and optimizing its parameters is a critical step in this process [33,34,43]. The results of these previous studies and the current aims are summarized in Figure 1. In the current study, an ultrafast Ti:Sa laser is used for marking for the first time. This aims firstly to enhance the speed of the marking and to increase the possibility of its future application in pharmaceutical industries. Furthermore, two different sizes/types of QR codes are marked on the surface of double film-coated ibuprofen tablets to determine data capacity, e.g., how much information can be encoded into a QR code using this type of marking method. Also, there are concerns that this laser-based QR code marking is damaging functional coatings or active tablet cores. Therefore, the laser effects on physical, chemical, and microscopic properties of this dosage form are evaluated under different processing conditions.

Additionally, despite TiO_2_’s proven track record in various industries, not just pharmaceuticals, its use in the last same field has received considerable criticism, especially after its ban on food by the European Food Safety Authority (EFSA) under Regulation 63/2022. Moreover, the European Commission (EC) asked pharmaceutical companies to evaluate several potential alternatives to this excipient (many TF coating formulations) without compromising the safety, quality, efficacy, and availability of the medicines concerned [53,54,55,56]. However, in August 2025, the European Commission issued a decision stipulating the necessity of maintaining this excipient as a pigment/opacifier in medicinal products, given the numerous challenges outlined in the literature if its serious removal were to occur. EMA also suggests that pharmaceutical industries should keep abreast of and adapt to new scientific developments regarding excipients, particularly when developing new products. This ensures that marketing authorization applications for new drugs have sound justifications for their choice of excipients, including the use of TiO_2_ [57].

Therefore, another objective of this study was to compare the physicochemical properties of the examined coatings (TC and TF). This comparison is important to evaluate the performance of one of the currently proposed alternatives to TiO_2_, as the search for promising substitutes for this excipient remains a highly relevant topic in pharmaceutical technology.

## 2. Materials and Methods

### 2.1. Materials

The composition of the tablet cores was as follows: ibuprofen (Ibuprofen DC 85, BASF, Ludwigshafen, Germany) as the model API; microcrystalline cellulose (Vivapur^®^ 102, JRS Pharma, Rosenberg, Germany) as a filler; dry binder, crospovidone (Kollidon^®^ CL-M, BASF, Ludwigshafen Germany), as a disintegrating agent; magnesium stearate (Molar Chemicals, Halásztelek, Hungary) as lubricant and anti-adhesion agent; and talc (Molar Chemicals, Halásztelek Hungary) as a glidant at concentrations of 16.66%, 74.33%, 5%, 1%, and 3%, respectively.

Two different ready-to-use commercial formulations were used after dispersion with distilled water to achieve a double-film coating. The first layer was an enteric coating based on a poly(methacrylic acid, ethyl acrylate) in a 1:1 ratio (Acryl-EZE^®^ MP 93018508 White, Colorcon, Idstein, Germany). The second layer was based on non-ionic hydroxy-propyl-methyl-cellulose polymer differently colored with or without TiO_2_ (Opadry^®^ 03F205045 Blue (TC Blue), 265F205024 (TF Blue), Opadry^®^ 03F265034 Brown (TC Brown), and 265F265001 (TF Brown) (Colorcon, Budapest, Hungary) to enable visual contrast after marking. All materials were used as received.

### 2.2. Direct Compression

Ibuprofen tablets were prepared using the direct compression method. A homogeneous mixture of drug, filler, and disintegrant was prepared in a Turbula mixer at 50 rpm for 8 min (Willy A. Bachofen Maschienenfabrik, Muttenz, Switzerland). The lubricant and glidant were then added and mixed for an additional 2 min. The final mixture was then compressed into round, slightly concave tablets with a mass of 300 mg and a diameter of 10 mm using a Korsch EK0 single-punch eccentric tablet press (E. Korsch Maschienenfabrik, Berlin, Germany). The targeted hardness of tablets was 80–100 N, which was achieved with 9 mm filling and 3 mm of immersion depth.

### 2.3. Film Coating

In this research, several types of aqueous film coatings were prepared according to the manufacturer’s recommendations. In the case of the aqueous dispersion of Acryl-EZE^®^ MP, the solid content was 20% *w*/*w*, while for the various Opadry^®^ batches, it was 15% *w*/*w*. The coating powders were gradually added to distilled water, initially stirred rapidly, and followed by a slightly slower rotation speed for 20 or 45 min, respectively, at ambient temperature. After preparation, the coating dispersions were sieved through a 250 micron mesh prior to the coating process to eliminate aggregates. To maintain homogeneity, gentle stirring was applied throughout the coating process. Film coating was carried out in batches of 400 g tablets in a 4M8 Pan coat (ProCepT, Zele, Belgium) perforated coating pan under specific conditions as recommended by the suppliers for each type of coating. A 0.5 mm spray nozzle was used to apply the atomized spray coating dispersion. The atomizing air pressure and the airflow rate were 2.0 bars and 0.70 m^3^/min, respectively. The drying and cooling process lasted 15 min. Additional coating parameters are summarized in Table 1 and Table 2 for Acryl-EZE^®^ MP and Opadry^®^ (TC and TF) coatings, respectively.

### 2.4. 3D Printing of the Tablet Holder

**Figure 2:**
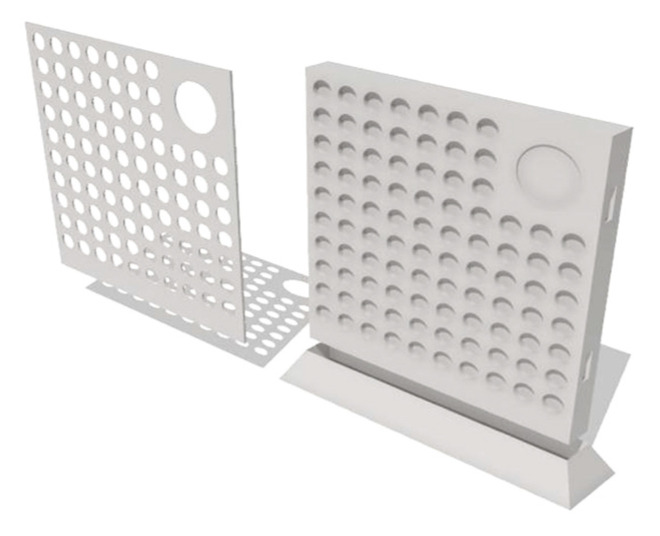
The 3D design of the tablet holder and the lid printed using FDM technology.

A specific tablet holder and lid were prepared using fused deposition modeling (FDM) 3DP technology. The design of the holder and lid was based on the geometry of the tablets and the laser settings. Figure 2 presents the 3D model of the printed components.

Shapr3D CAD software (Shapr3D Zrt., Budapest, Hungary; version: 5.370) was used to design the shape of these objects, which was exported in high-resolution STL format and then sliced using Craftware (CraftUnique, Budapest, Hungary; version: 1.23). The objects were printed separately in two print plates using a CraftBot Plus Pro 3D printer (CraftUnique, Budapest, Hungary) with the following parameters: 0.2 mm layer height, 0.4 mm nozzle, 210 °C hotend temperature, 60 °C bed temperature, and 80 mm/s feed rate. The polymer used in this study was polylactic acid (PLA) filament (Herz Hungária Ltd., Üllő, Hungary).

### 2.5. Laser Ablation of Coated Tablets

**Figure 3:**
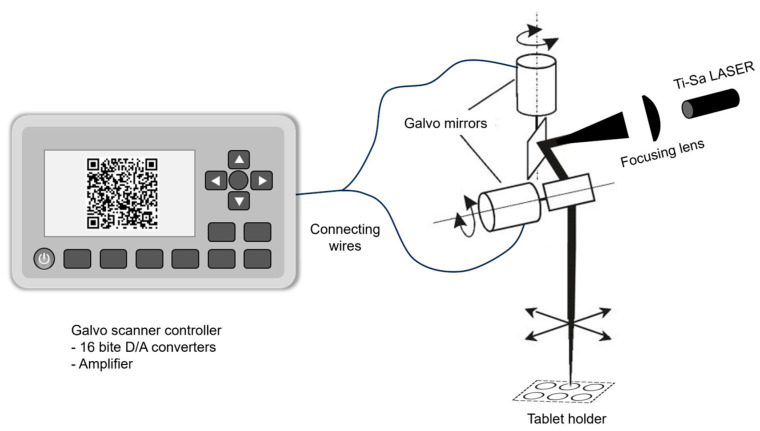
The experimental setup for the laser processing.

Double-coated ibuprofen tablets were irradiated by a titanium-sapphire (Ti:Sa) chirped pulse amplification (CPA) laser (ELI-ALPS Research Institute, Szeged, Hungary). The laser system provides 92 fs, 4 mJ laser pulses at 1 kHz repetition rate and a central wavelength of 781 nm. The system consists of an erbium-doped fiber oscillator (C-fiber, Menlo Systems, Martinsried, Germany), an Öffner-stretcher, a regenerative amplifier, a multipass amplifier, and a grating compressor. To be able to make the QR code, the beam needed to be moved with a help of a galvo mirror according to the experimental setup shown in Figure 3.

In this study, various laser pulse energies (200–400 µJ) and numbers of pulses (20–240) were applied as factors to evaluate their effects on the readability of the QR code and the coating stability. The laser fluence (F) at the tablet surface can be calculated as

F = E/πr^2^

where *E* is the pulse energy (200–400 µJ) and *r* is the beam radius at the focal plane. Based on the focused spot diameter of ~100 µm (measured by knife-edge method at 781 nm), the calculated fluence ranged from 2.55 to 5.09 J/cm^2^ across the experimental parameter space. The range of the used laser scanning speed was between 0.2–2.5 mm/s.

### 2.6. Physical Properties of Tablets

The dimensions of 20 randomly selected tablets were measured using a digital caliper (VWR Traceable^®^, Bengaluru, India). The uncoated and coated tablets (n = 20) were weighed using an analytical scale, and the hardness (H) of 10 tablets of each formulation was measured using a manual tablet hardness tester (SOTAX MT50, Aesch, Switzerland). Additionally, tablet friability (F) was measured by a friability tester (Erweka, Langen, Germany) before and after the coating process. The test was carried out by placing approximately 6.5 g of each formulation according to European Pharmacopoeia (Ph. Eur.) 11th Edition (general chapter 2.9.7 “Friability of uncoated tablets”) in the device and rotating 100 times. The percentage of weight loss (%) was then calculated. Mechanical properties (H and F) were also applied to the laser-treated tablets.

The residual water amount of the uncoated and coated tablets was investigated with a moisture analyzer (RADWAG MAC 50/NH, Radom, Poland) based on the loss-on-drying method. This test was carried out three times (n = 10) for each formulation after storage in plastic containers. The duration of the test was 8 min, and the maximum temperature was 105 °C.

### 2.7. Coating Thickness and Weight Gain (WG)

The final coating thickness was measured on tablet cross sections using a stereomicroscope (Zeiss, Oberkochen, Germany) at a magnification of ×5.4. Tablets from each formulation were cut in half along the middle of the tablet band, and each coating thickness was measured at a minimum of 10 points (n = 10 per half). Then, the average thickness was calculated [34]. The WG test is commonly used to determine the amount of coating produced by determining the weight difference between a specified number of tablets. In total, 20 tablets of each formulation were weighed before and after the coating, and the WG was calculated using the formula: WG% = [(final weight − initial weight)/initial weight] × 100 [58].

### 2.8. In Vitro Disintegration Test

Unlasered and lasered film enteric-coated ibuprofen tablets were tested using a disintegration apparatus (Erweka ZT 71, Erweka GmbH, Langen, Germany) to determine the disintegration time (DT) according to Ph. Eur., 11th Edition (general chapter 2.9.1 “Disintegration of tablets and capsules”). First, the tablets were placed in 900 mL of artificial, enzyme-free gastric juice (pH = 1.22) for 2 h and then in phosphate buffer (pH = 6.82) for approximately 1 h. The pH values were checked with a pH meter (Hanna Instruments, Smithfield, RI, USA). The test was carried out on six tablets of each formulation at 37 ± 0.5 °C. In addition, the uncoated tablets were also tested under the same conditions as above, but in distilled water for 15 min.

### 2.9. In Vitro Drug Dissolution Test

This study was conducted on the laser-treated (readable under the minimum laser parameters and gastro-resistant) and unlasered tablets using a dissolution tester (Erweka DT 700, Erweka GmbH, Langen, Germany). This test was carried out according to Ph. Eur., 11th Edition (general chapter 2.9.3 “Dissolution test for solid dosage forms”), using the rotating basket method at a rotation speed of 100 rpm. The dissolution medium was 900 mL of pH 1.22 artificial enzyme-free gastric juice, which was replaced by 900 mL of pH 6.82 after 2 h. The dissolution medium was maintained at 37 ± 0.5 °C. Six tablets of each formulation were tested. Samples of the dissolution medium (5 mL) were drawn and mechanically filtered at different time intervals for each medium: 30, 60, 90, and 120 min for the gastric medium and 5, 10, 15, 30, 45, and 60 min for the phosphate buffer. It should be noted that the drawn samples were not replaced and the loss of medium was considered in the calculations. The absorbance of these samples was measured at 222 nm using a spectrophotometer (Genesys 10S UV–VIS, ThermoFisher Scientific Inc., Waltham, MA, USA). The results were expressed as the cumulative percentage of the drug released over time.

### 2.10. Data Capacity by QR Code Version and Size

This study aimed to determine how much information can be accurately encoded on the surface of a tablet of a given size using laser ablation. To better understand the limitations and possibilities of this type of marking method, it is important to examine how much information can be encoded into a QR code. The amount of storable data depends primarily on the size (version) of the QR code and the level of error correction applied. As the data volume increases or higher error correction is used, the matrix size must also grow accordingly [59,60]. QR codes come in 40 different versions, ranging from Version 1 (21 × 21 modules) to Version 40 (177 × 177 modules). The more characters are stored, the larger the version that is needed. Table 3 shows how these factors interact in some versions. (Note: Values shown are for alphanumeric characters (0–9, A–Z, space, and nine symbols.) Numeric-only data can store 1.8× more characters; byte data stores fewer) [61].

#### Rationale for QR Code Size Selection

The physical dimensions and version (matrix size) of the QR codes were selected based on three interdependent constraints:

1. Tablet geometry: With a diameter of 10 mm and a slightly concave surface, the useful marking area is limited to approximately 6 × 6 mm to avoid edge effects and curvature-induced distortion during smartphone scanning. Codes exceeding this size would extend onto the tablet band, reducing readability [43].
2. Laser and optical resolution: The galvo scanning system (Figure 2) has a positional accuracy of ±5 µm and a focused spot size of ~100 µm (781 nm wavelength). For reliable smartphone camera detection (typical resolution 1–2 megapixels at 10 cm distance), individual QR modules must be ≥200 µm. The selected versions and sizes have different advantages. Version 1 at a size of 5 × 5 mm has a module size of 238 µm and is associated with optimal balance of readability and information density. Version 1, at a size of 6 × 6 mm, has a module size of 286 µm, offers enhanced contrast, and was tested for comparison. Version 3, at a size of 5 × 5 mm, has a module size of 172 µm, enables maximum density achievable within resolution limits, and was tested to assess scalability for extended data (e.g., ePIL URLs and patient identifiers).
3. Pharmaceutical data requirements: Industry guidelines indicate that tablet-level QR marking by laser is considered a complement and a replacement of legally mandated pack-level serialization (EU Directive 2011/62/EU, Commission Delegated Regulation 2016/161 [44,45]). Similar serialization requirements exist in the US under the Drug Supply Chain Security Act (DSCSA), which mandates unit-level traceability by 2023. DSCSA outlines steps to achieve an interoperable and electronic way to identify and trace certain prescription drugs at the package level as they move through the supply chain [62]. In this context, tablet-level serialization [39,46], particularly used for small, high-value batches and hospital/clinical personalization, must contain essential information (API name, dose, batch number, and classification), which requires 15–35 alphanumeric characters. This is enabled by QR Version 1 at medium error correction, which encodes 20 characters. Nevertheless, the 61 characters encoded by Version 3 enables future integration with electronic health records or supply-chain databases [42,52] or may be suitable to code simple URL for information leaflets. In present study, it was used to code the following text: “Ibuprofen-50 mg-3x-NSAIDs-SZTE.”

These criteria guided the selection of the two test conditions (5 × 5 mm numeric vs. alphanumeric codes, Version 1 vs. Version 3) to evaluate both baseline feasibility and maximum information capacity within the tablet surface constraints. The laser ablation was optimized for QR Code Version 1. However, Version 3 was also tested. Therefore, two different datasets were encoded: a numeric one and a larger alphanumeric one.

### 2.11. QR Code Readability by the Smartphone

By removing the top coating layer of the tablets using laser ablation, the QR codes become readable, making them suitable for distinguishing genuine drugs from counterfeit ones. A suitable QR code scanner application (e.g., QRbot) can be downloaded from the Internet and used on any patient’s smartphone. Furthermore, unlike the usual color of the QR code, the current one is white, while the tablets are colored. Therefore, the downloaded application should be able to read the code in reverse [43].

### 2.12. Profilometry Measurements

The ablation depth was determined by contact profilometry (Veeco Dektak 8 Advanced Development Profiler^®^, Veeco, Plainview, NY, USA) taking cross-sectional profile readings at various points on the QR codes produced by laser irradiation. Measurement conditions were as follows: the radius of curvature of the used tips: ≈2.5 μm, cone angle: 45°, force applied to the surface during scanning: 29.4 Μn, scan length: 2000 µm, vertical resolution: 40 Å, and horizontal range between 0.1–0.13 μm. Data were evaluated using Vision^®^ 32 software for data analysis.

### 2.13. Scanning Electron Microscopy (SEM)

The morphology of the laser-treated tablet surfaces was investigated by scanning electron microscopy (SEM) (Hitachi S-4700, Tokyo, Japan) using 10 Kv acceleration voltage. The tablets were fixed to the sample holder using a double-sided carbon adhesive tape, and then, a very thin layer of conductive gold was sputtered onto them by the sputter coater Quorum Q150R S plus (Quorum Technologies Ltd., Laughton, UK).

### 2.14. Raman Microscopy

Raman spectroscopic measurements were conducted to investigate tablets subjected to laser treatment (readable under the minimum laser parameters and gastro-resistant). The spectra were acquired using a ThermoFisher DXR Dispersive Raman spectrometer (ThermoFisher Scientific Inc., Waltham, MA, USA), equipped with a CCD detector and a diode laser operating at a wavelength of 780 nm. Measurements were performed at laser powers of 12 Mw, with a slit aperture size of 25 μm.

Spectra were collected for both untreated tablets and those exposed to laser treatments, using an exposure time of 6 s per acquisition. The spectral range covered was 1800–200 cm^−1^, and automated fluorescence correction was applied during the measurements. Data was evaluated with Spectragryph v. 1.2 optical spectroscopy software [63]. Each spectrum represents the average of 12 individual scans.

**Figure 4:**
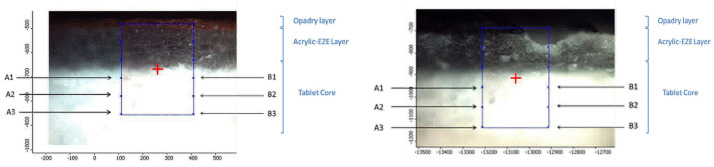
Sampling locations for Raman spectroscopy on the halved tablet surface before and after the laser processing.

Three differently coated tablets (Opadry^®^ TC Blue, TC, and TF Brown) were examined by Raman spectroscopy before and after laser treatment. Spectral data were collected from the halved surface of the tablets (Figure 4).

The Opadry^®^ and Acryl-EZE^®^ MP coating layers and the tablet core are indicated. The left panel shows the cross section of the intact (non-lasered) tablet, while the right panel displays the cross section of a laser-marked tablet with the ablation hole of the upper coating layer clearly visible. Raman spectra were collected from six positions in the core. From laser-treated tablet (right), three spectra were collected (A1–A3) from the points aligned in a row adjacent to the ablation zone and three spectra from points (B1–B3) next to it, located directly beneath the ablation area.

### 2.15. Statistical Analysis

The results of most tests are expressed as mean ± standard deviation using Microsoft Excel 2021 software. Statistical analysis was performed using Student’s *t*-test for the comparisons of two groups and one-factor ANOVA for the comparisons of more than two groups. Differences with *p* < 0.05 were considered statistically significant.

## 3. Results and Discussion

### 3.1. Physical Properties of Uncoated and Coated Tablets Before Laser Processing

Regarding appearance, the prepared tablet cores were white, slightly concave, round, without score lines, uniform in size, smooth in texture, and without any visible defects. The nominal mass was 300 mg, with a thickness of 3.235 mm ± 0.02 and a diameter of 10.00 ± 0.01 mm. The tablet mass uniformity complied with the standards accepted in the Ph. Eur. (±5% for tablets ≥ 250 mg).

Table 4 shows the main physicochemical properties of uncoated and coated (TC and TF) tablets before laser treatment for easier comparison. These formulations met the USP requirements regarding the DT of immediate (within 15 min) and delayed release tablets [64]. The DTs are listed in the same table. Represent the values for these formulations in phosphate buffer, and it can be observed that they do not differ significantly from each other. The uncoated and coated tablets also showed good attrition resistance as weight loss did not exceed 1% in friability test [9].

Hardness values increased in the presence of the coating layer and appeared to increase more with the application of an additional layer. On the other hand, the moisture content decreased after coating in the same manner, which could be due to the presence of a polymeric barrier around the tablet core that reduces adsorption of external moisture and increases the mechanical resistance during handling. The hardness has increased by approximately 50–140% upon coating. However, this increase depends on the nature, composition, and thickness of the coating material used [65,66,67]. The moisture content of the core tablets was 4%, which is in good accordance with what is indicated as acceptable for microcrystalline cellulose tablets (should not exceed 5%) by the literature [65,68,69], which was further decreased due to drying after the coating process, which is considered to enable suitable microbiological stability.

### 3.2. Comparison of TC and TF Coatings

Based on the data presented in Table 4, no significant differences (*p* ˃ 0.05) were observed in the tested physical properties of Opadry^®^ TC and TF coated tablets in both colors (blue and brown).

The issue of omitting TiO_2_ has grown in importance considering recent developments. Several research papers have evaluated the previous properties of some commercial coatings that are completely free of TiO_2_. For instance, Galata et al. compared the performance of TC and TF coatings through a range of physical properties similar to those assessed in this study. They found that the removal of this excipient did not significantly affect moisture absorption if the coating thickness was sufficient. The same has happened with friability and dissolution. The results of this study and others [70,71] were consistent with our findings.

### 3.3. WG and Coating Thickness

One of the most important indicators to measure after coating is the weight gain (%), which indicates the amount of coating components deposited on the surface [72,73]. The weight gain of TF Blue, TC Blue, TF Brown, and TC Brown formulations was 12.49 ± 0.01%, 9.65% ± 0.02%, 6.58 ± 0.02%, and 5.25 ± 0.02%, respectively. These values can vary depending on the coating requirements and type. From a manufacturing perspective, it is generally considered acceptable within 8–12% regarding enteric coating. However, excessive weight gain can alter the DT or even the drug release pattern [74,75].

In this study, the coating process was stopped once the complete coverage of the tablets had been achieved. The difference between blue and brown coatings can be due to the different opacifying ability of different formulations. Although the basic composition is almost the same, the amount and/or type of pigments is strongly different [76]. The Opadry^®^ TC Brown coatings commonly contain the mixture of black iron oxides and TiO_2_ as pigments; meanwhile, the mixture of TiO_2_ and FD & C Blue No. 2 is the usual one in case of blue coatings from the same manufacturer [77]. In addition, current TF coatings often use calcium carbonate (CaCO_3_) as an alternative opacifier to TiO_2_. It is known that CaCO_3_ exhibits a lower opacity level than TiO_2_, which requires the application of a larger amount to achieve the desired appearance [53,76,77].

**Figure 5:**
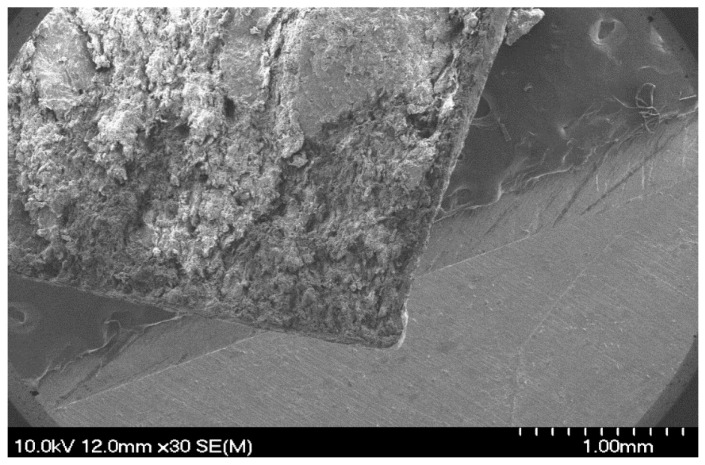
SEM micrograph of the Opadry^®^ TC Brown coated tablet’s cross-section surface at a magnification of 30×.

Furthermore, both layers in these formulations were approximately 50–60 µm thick for all coating types (Figure 5).

The literature data indicate that the required coating thickness to protect tablets from gastric acid is approximately 30–50 µm. However, some formulations may require higher coating thicknesses (≥100 µm), depending on drug, polymer type, coating consistency, and tablet size. Moreover, a balance should be maintained between gastric protection and dissolution profile in the target intestinal region [78].

### 3.4. QR Code Results

#### 3.4.1. QR Code Marking Speed and Precision Enhancement

Track-and-trace obligations exist across major markets; accordingly, the tablet-level QR marking by laser is not intended to replace legally mandated pack-level serialization, but to complement it in specific scenarios. In this study, laser ablation of QR codes on coated tablets required 1.5–7 s per unit, depending on the parameter settings, corresponding to approximately 514–2400 tablets per hour for a single-head offline station. Considering that previous studies reported that the marking times of 2 h with argon fluoride- (ArF) and 10 min with femtosecond lasers [33,43], the first objective, to demonstrate speed and precision enhancement sufficient for smaller production batches, was achieved. At the demonstrated rates (1.5–7 s per unit; ≈514–2400 tablets/h), a dedicated offline station could already support small clinical or hospital-pharmacy use, for example, ward- or patient-specific personalization of doses and post-manufacturing marking of small, high-value batches to reinforce anti-counterfeiting and enable targeted follow-up. For larger commercial lots, parallelization (multi-head systems/lanes) would be required to approach typical industrial throughputs.

Additionally, the stability of the materials used under the applied processing conditions is expected to remain unaffected, given that the laser-material interaction time is extremely short (as confirmed by Raman analysis). Despite the high wavelength range of the laser used (near IR, NIR), its repetition rate is high (1 kHz), which could enable the required marking speed without impacting the accuracy of the code and the safety of pharmaceuticals. In other words, both thermal impact and chemical damage during materials removal will be negligible if laser parameters are controlled correctly. Furthermore, this type of laser can create magnificent structures and is considered precisely adjustable in a clean way [43,79,80].

Moreover, due to its high energy, this type of laser is able to remove TiO_2_ particles from TC coatings [43]. This will avoid problems related to the formation of TiO_2_-black particles and the resulted unrecognition and color changing of the QR code from white to black or dark blue due to oxygen vacancy of TiO_2_ [31,33,43,81,82], or as was concluded by Matsushima et al. due to photocatalytic degradation of the coating materials upon irradiation of TiO_2_, which could result in a significant change in the color and appearance of the coated tablets [83]. Indeed, this problem was not observed in the studied coatings, whether TC or TF, after treatment with this laser.

#### 3.4.2. QR Code Applicability

**Figure 6:**
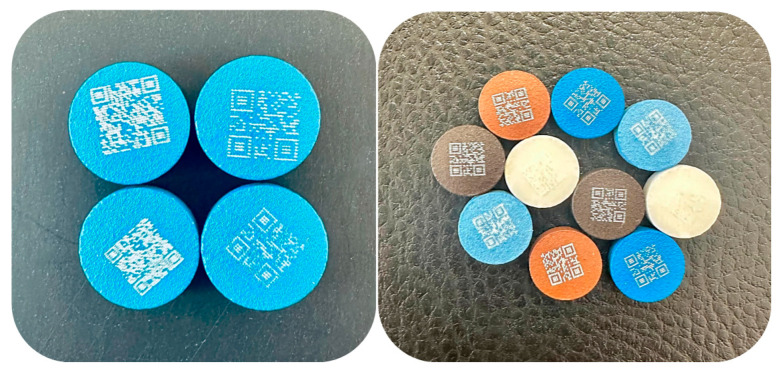
Different types of QR codes ablated on ibuprofen-coated tablets (**right**) and Opadry^®^ TC Blue coated tablets (**left**).

The secondary objective of this work was to evaluate the amount of information that could be encoded into a QR code using this marking method. Two kinds of information of varying length and character composition were encoded into QR codes of different sizes (Figure 6). Subsequently, the impact of marking resolution on QR code readability was evaluated. This parameter refers to the density of laser-ablated points forming the QR pattern, which may affect how accurately the code can be interpreted by smartphone cameras. The simpler code (QR Code Version 1) was numeric, 12345678, while the more complicated code was alphanumeric (QR Code Version 3), Ibuprofen-50 mg-tablet-3x-NSAIDs-SZTE. This information content is considered enough in terms of drug security, as it is larger than the number of characters available in traditional barcodes [84]. Each of these two codes (numerical and alphanumeric) was designed in two dimensions: 5 × 5 mm and 6 × 6 mm. The QR code was formed from round, partly overlapping ablated holes and was valid and readable even in the cases of dotted parts or when the beam was not perfectly centered on the surface. The last cases might be explained by the ability of the QR code to correct errors and restore lost data [43,48].

#### 3.4.3. QR Code Readability

Based on the results displayed in Table S1 (Supplementary Materials), it can be observed that the readability of QR code differs depending on the type and color of the used coating, as well as the applied processing parameters. For example, QR code readability with Opadry^®^ TC Brown and TF Brown coatings was high under most of the conditions in which they were tested. However, it was very difficult to notice the code with Opadry^®^ TF Blue coating due to its very light color. Meanwhile, the darker color of TC Blue coating enabled readability, but it was more difficult to find the appropriate combination of these parameters to achieve it compared to brown coatings.

This difference in readability can be explained by several factors related to physical and optical properties of the previous coatings, such as color contrast, surface reflectivity or gloss, coating thickness, and homogeneity, as well as the interaction with the laser (e.g., absorption, diffusion, scattering, etc.) [33,43,85,86]. In addition, the code also becomes more readable as the laser pulse energy or the number of pulses used increases. However, the above conditions should not be extreme as they could negatively impact the functional coating, the API, or the dosage form [43].

Beside the lack of CR code readability, the coverage capacity of the Opadry^®^ TF Blue coating was very poor, as the tablets were unequal in color, so based on these results, this color was excluded from further studies.

For other coatings, readable tablets marked at the lowest pulse energy (245 µJ) and number of laser pulses (30, 35, and 80 for Opadry^®^ TC Brown, TF Brown, and TC Blue coatings, respectively) were used in further investigations, unless indicated otherwise.

It should be noted that the study was designed as a formulation-specific parameter optimization rather than a head-to-head comparison under identical laser settings. Parameters were adapted by design: once QR code readability was achieved for a coating, we did not escalate further, while other coatings required extended exploration. Our conclusions are restricted to feasibility and to the coating-specific parameter windows that enable robust readability rather than ranking coatings against each other. Future work will include controlled inter-coating comparisons under matched parameter sets.

### 3.5. Surface Profilometery Measurements

Surface profilometry is a non-invasive and highly accurate technique for the quantitative characterization of laser ablation depth and changes in surface roughness [30,32,41,49,50,51].

**Figure 7:**
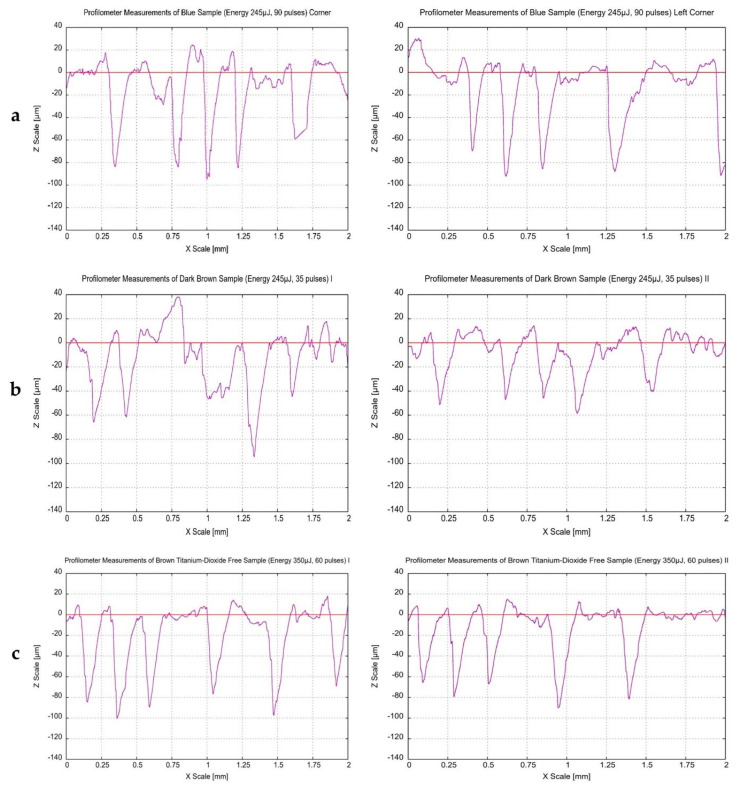
Profilometry line scans across the laser ablation holes created on the surface of (**a**) Opadry^®^ TC Blue, (**b**) TC Brown, and (**c**) TF Brown coated tablets, taken at two different positions within the QR code area at (245 µJ, 90), (245 µJ, 35), and (350 µJ, 60), respectively.

Line-scan surface profiles of the Opadry^®^ TC Blue, TC Brown, and TF Brown coatings are presented in Figure 7. It was found that the ablation depths were similar in different areas of the QR codes, such as the center (I), edges (II), or corners, for all types of coatings. Some studies have indicated that the consistency of coating thickness and its spatial distribution on the surface can be assessed by comparing different ablation depth values at multiple locations on the same tablet [87,88], which is consistent with our current findings.

**Figure 8:**
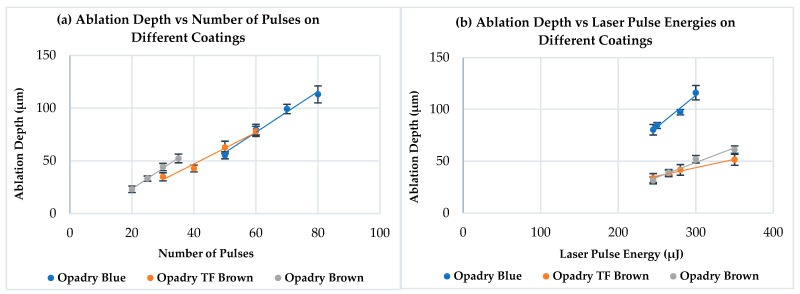
Ablation depth as a function of (**a**) the number of laser pulses and (**b**) the laser pulse energy in case of Opadry^®^ TC Blue, TF Brown, and TC Brown coated tablet surface, respectively (n = 3).

The results of ablation by the ultrafast Ti:Sa laser for different coatings are highlighted in Figure 8. There is a linear relationship between the ablation depth and the number of laser pulses used, which confirmed similar findings of other research papers [34,43,89]. Furthermore, the same relation was observed between the ablation depth and the applied laser pulse energy in this research. It can also be observed in Figure 8a (different energies and constant number of pulses) that the Opadry^®^ TC Brown coating exhibited slightly higher ablation depths than those related to the TF Brown one. This can be possibly attributed to the differences in their compositions (primarily pigments) and their intrinsic physical properties, which probably affect the interaction behavior with this laser. However, this difference was not significant (*p* > 0.05).

The reactions of TiO_2_ to laser ablation differ significantly from those of other ingredients in the coatings. Each material has a characteristic ablation threshold, which is specific to the material, laser type, ablation method, and fluence. In case of TiO_2_, three main events are expected to occur apart from ablation as result of laser treatment: reduction, phase transition, and melting. These can be qualitatively classified according to the respective characteristic temperatures of 500 °C, 750 °C, and 1870 °C [33]. Photon–TiO_2_ interactions mainly involve photon absorption, where photons with energy above TiO_2_’s bandgap (~3.0–3.2 Ev) are absorbed, generating electron-hole pairs that profoundly alter the optical properties through excitation, recombination, and defect-mediated processes [90]. Regarding ultra femtosecond laser, pulses induce multiphoton absorption in TiO_2_ films, generating electron-hole pairs that drive photochemical oxidation/reduction or micro-nanostructures without significant heat, altering optical properties like reflectance and bandgap for enhanced light trapping. This creates color marks via ablation followed by H_2_O_2_ annealing, yielding durable contrasts ideal for pharmaceutical traceability [91].

Detailed comparison between Opadry^®^ TC Blue and Brown (TC or TF) coatings or even the application of the previous observations on Figure 8b is not possible due to the varying conditions (different number of pulses and energies).

The benefit of determining the ablation depth is to fine-tune the laser processing parameters to achieve the desired penetration without causing any damage to the API, dosage form, or even functional coatings [92,93,94,95].

**Figure 9:**
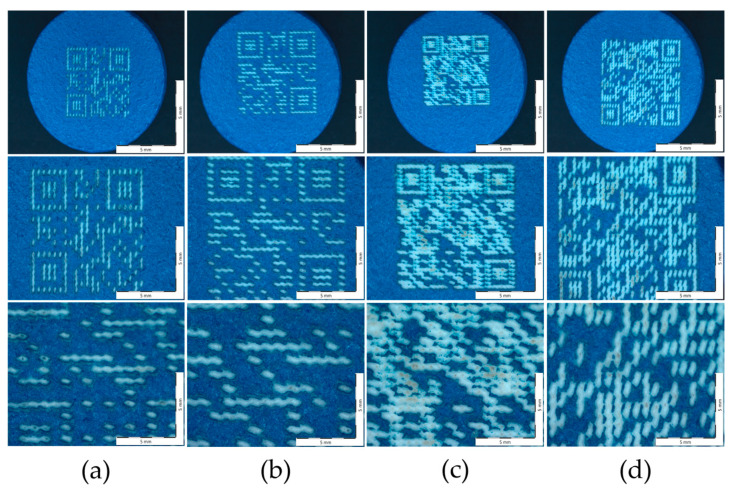
Surface morphology images of Opadry^®^ TC Blue coated tablet treated by ultrafast Ti:Sa laser in case of (**a**) 5 × 5 numeric QR code, (**b**) 6 × 6 numeric QR code, (**c**) 5 × 5 alphanumeric QR code, and (**d**) 6 × 6 alphanumeric QR code at 0.65×, 1.25×, and 2.5× magnifications, from top to bottom, respectively.

Figure 9a–d shows the morphological images taken of four different QR code patterns on the surface of Opadry^®^ TC Blue coated tablets using a Zeiss stereomicroscope at magnifications of 0.65×, 1.25×, and 2.5× (from top to bottom, respectively). Varying the QR code size/type did not result in any differences in the above values for the same parameters (*p* ˃ 0.05).

Additionally, the ablated areas (holes) on the surface appear to have a square or rectangular shape. Each formed hole can be described as a depression with clear contours, relatively straight edges, and right angles. These holes are also arranged in a regular, repetitive grid, or matrix pattern. This arrangement suggests that the ablation was highly precise because of the application of a precisely programmed laser. Furthermore, the edges of these holes also appear sharp and well-defined, with little evidence of melting or irregularity, supporting the accuracy of the ablation process. Moreover, the ablated areas are also clearly visible, appearing to be different in color than the untreated surface regions.

Higher magnification (2.5×) reveals that the ablated parts are elongated and have parallel grooves, with sub-patterns resembling barcodes or pixels within each hole (Figure 9a,b). However, it is well visible (Figure 9c,d) the increased amount of information within the code, especially without increasing the size (Figure 9c), resulted in overlapping and irregular-shaped ablation areas. Furthermore, the surface features became less distinct, and the areas of the non-lasered regions also decreased compared to the other images.

### 3.6. SEM Imaging

**Figure 10:**
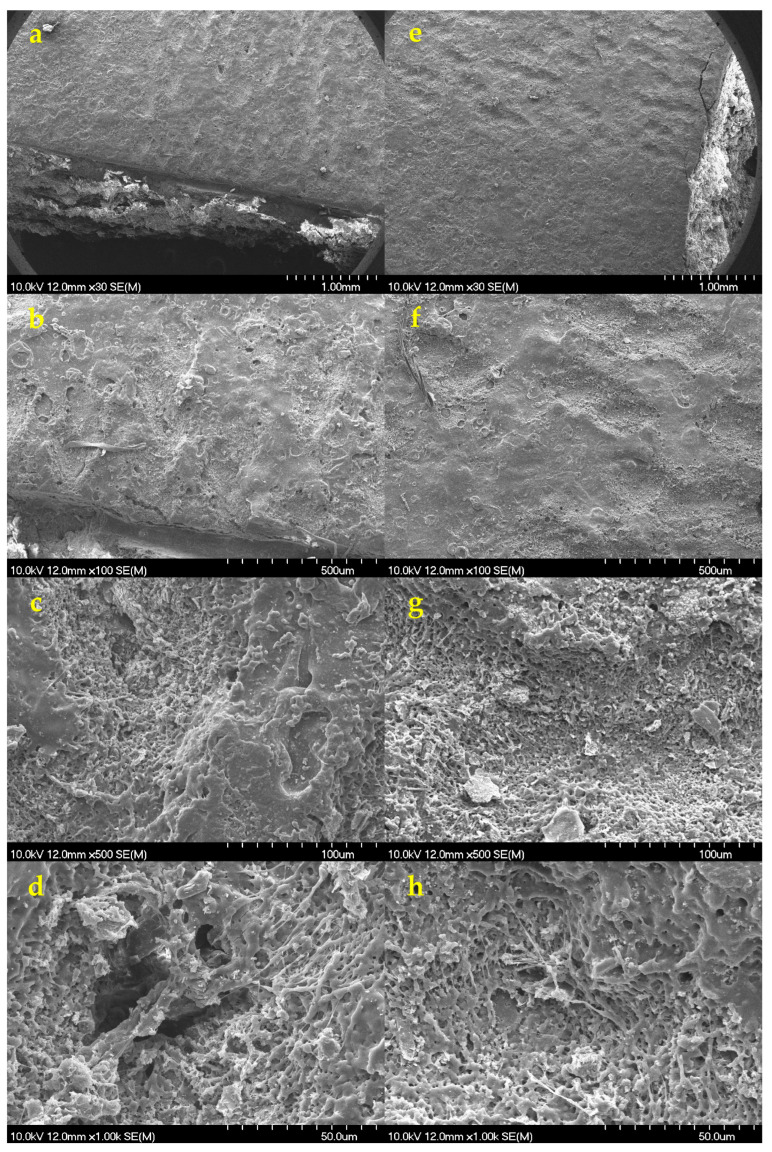
SEM micrographs of the laser-treated surfaces of (**a**–**d**) Opadry^®^ TC Brown and (**e**–**h**) Opadry^®^ TF Brown coated tablet at 30×, 100×, 500×, and 1000× magnifications (zooming in from top to bottom on the ablated region).

To further characterize the laser-treated regions, SEM images were taken to provide detailed information on their surface morphology and the physical changes that had occurred. Figure 10 displays the laser-ablated (245 µJ and N = 35) surface of Opadry^®^ TC Brown (Figure 10a–d) and TF Brown (Figure 10e–h) coatings for comparison.

Figure 10a,e show top views of a QR code on a halved tablet and confirm that the QR code is made up of overlapping craters, as described above. The untreated surface is clearly smooth and uniform, but traces of some coating errors (e.g., bubble formation) can be identified despite them being invisible to the naked eye. The ablated surface is highly porous and uneven.

Figure 10b,f display the treated surface at a greater magnification (100×), where intact regions can be identified between the holes. In addition, it appears that there is no significant difference between these two coatings in terms of the amount of damage (Figure 10c,g). This confirms the results of the surface profilometry. Figure 10d,h display the ablated hole at higher magnification. A different structure may be identified at the bottom of the ablation cavity (because of the melting), which is probably the gastro-resistant coating layer. This might indicate that the ultrafast Ti:Sa laser not only removed the upper coating, but also penetrated the functional layer.

**Figure 11:**
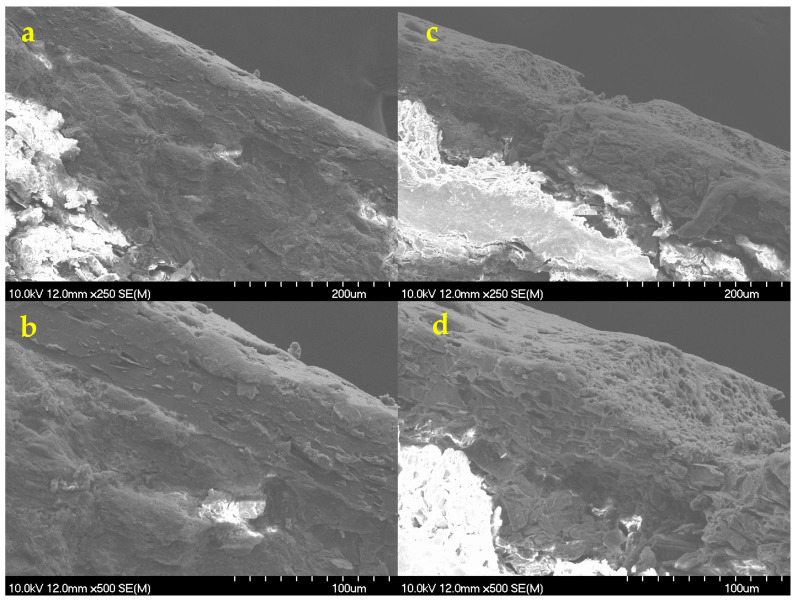
SEM micrographs of the tablet’s cross-section surface for Opadry^®^ TF Brown formulations in case of (**a**,**b**) before the laser processing and (**c**,**d**) after the laser processing at the same magnifications of 30×, 250×, and 500× (from top to bottom, respectively).

This was also confirmed by the scanning electron micrographs taken on the cross-sectional part of the same tablets (Figure 11).

The thickness of the double coatings was approx. 70 μm thick, depending on the position, while the thickness of the outer coating was only 20 μm. Considering the ablation depth, it can be confirmed that this thickness was insufficient to protect functional coatings and the core from the effects of laser processing. Since the originally intact structure of the coating became very loose and porous at the ablation area (Figure 11c,d), this might seriously affect the coating functionality, especially as most studies indicate the importance of achieving sufficient coating thickness in case of DR coatings [96,97].

### 3.7. Physical Properties of Film-Coated Tablets After Laser Ablation

The physical properties of the film-coated ibuprofen tablets were evaluated again after laser treatment to assess the effects of laser irradiation (Table 5). The H of tablets significantly decreased after laser irradiation (*p* < 0.05), while the F values remained statistically unchanged (*p* > 0.05). Laser ablation of coated tablets potentially induces local mechanical effects that weaken the coating structure, reducing the surface hardness of previous formulations [98]. Nevertheless, this type of treatment does not necessarily affect negatively the mechanical integrity of the tablet core, which is related to the friability (resistance to attrition). However, the surface properties and integrity (coating) are primarily affected [88], as the laser can also induce microscopic defects in the same structure, rendering it porous and brittle enough to allow fluid penetration and rapid disintegration [98], which was observed in present results, as that the tablets completely lost their gastro-resistance and disintegrated within a few seconds to a minute after laser treatment, which was indicated by the SEM measurements. The differences in DTs after the ablation were very small, considering that the tested formulations were processed at the same pulse energy (245 µJ) but with a different number of pulses (30, 35, and 80 for Opadry^®^ TC, TF Brown, and TC Blue, respectively).

### 3.8. Formulations with Increased Coating Thickness

To solve the problem of the change of physical integrity, new film-coated tablets were prepared under the same conditions, but with increased coating thickness. The properties of these tablets were re-evaluated before and after the laser processing.

#### 3.8.1. WG and Coating Thickness

The weight gain of these tablets was significantly higher (22.89 ± 0.02%, 23.26 ± 0.02%, and 30.45 ± 0.03%) for Opadry^®^ TC Blue, TC Brown, and TF Brown formulations, respectively. The similarity in weight gain values between the blue and brown coatings is purely coincidental, which was necessary to achieve sufficient thickness, but the difference between the Opadry^®^ TC Brown and TF Brown coatings to achieve complete coverage still exists.

The coating thickness of these formulations ranged between 200 and 250 µm, depending on the position. The functional layer became approximately five times thicker.

#### 3.8.2. QR Code Findings

The observations regarding the speed and precision of the marking process also apply to the tablets with increased coating. However, since the primary objective of these formulations was to improve the quality of the dosage form and make it suitable for laser processing, optimizing the data capacity of this code was not the focus here. Therefore, only QR Code Version 1 (5 × 5 mm) was marked on the surface.

Table S2 summarizes the laser parameters (both pulse energy and number of pulses) applied to these tablets, along with their readability results for each coating type. The increase in coating thickness may affect the readability of QR code, but it can be concluded that, similarly to the previous series, the Opadry^®^ TF Brown coating performed best, followed by the Opadry^®^ TC Blue and then the TC Brown ones. Readability also clearly increased with increasing laser pulse energy or the number of applied pulses.

The evaluation of characteristics followed the same scenario as earlier, e.g., tablets where readable QR code was achieved at the lowest laser pulse energy or number of pulses were tested.

#### 3.8.3. Surface Profilometry Measurements

The ablation depth values did not differ significantly whether QR code was located in the center of the tablet surface or slightly offset from it, if the same conditions were applied (*p* ˃ 0.05). In addition, the same applies to different locations of the treated region. The last result indicates the homogeneity of the coating layer distribution on the surface of these formulations as well.

**Figure 12:**
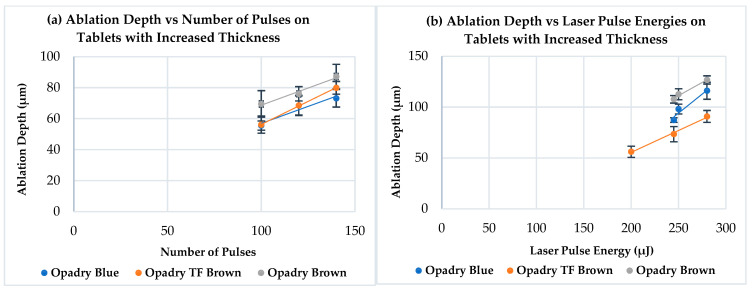
Ablation depth as a function of (**a**) the number of laser pulses and (**b**) the laser pulse energy in case of Opadry^®^ TC Blue, TF, and TC Brown coated tablet surface (with increased thickness), respectively (n = 3).

Furthermore, the same relationship was established between the ablation depth and the laser pulse energy or number of pulses (Figure 12), and no significant difference was observed between the tested tablets in terms of ablation depths under the same processing conditions (*p* ˃ 0.05). These observations are valid for all coatings studied.

#### 3.8.4. SEM Imaging

**Figure 13:**
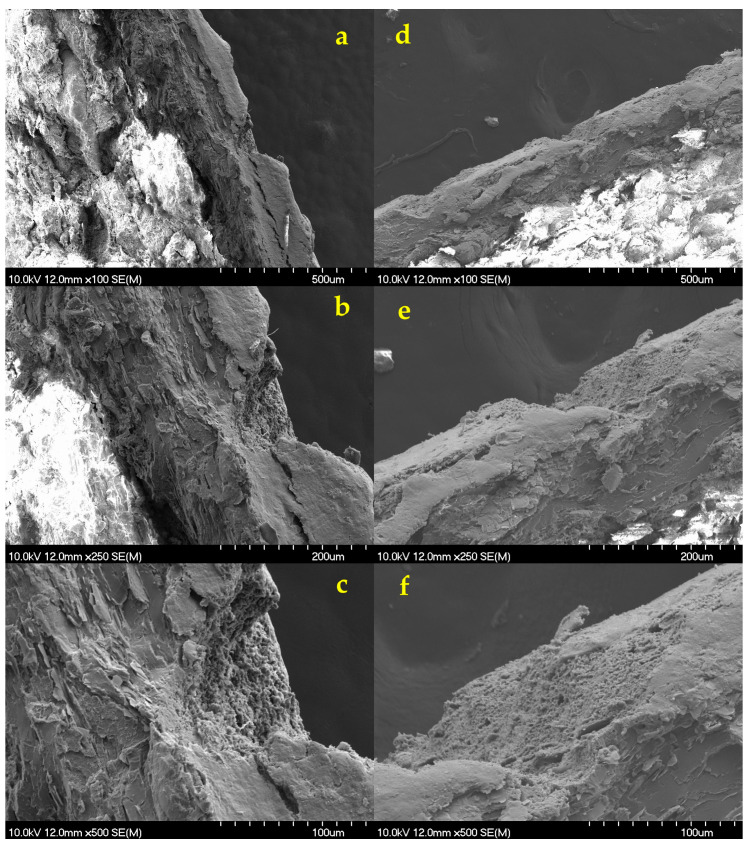
SEM micrographs of the laser-treated tablet’s cross-section surface for (**a**–**c**) Opadry^®^ TC Brown (280 µJ, N = 150) and (**d**–**f**) TF Brown (200 µJ, N = 140) coated tablets with increased thickness at the same magnifications of 100×, 250×, and 500× (from top to bottom), respectively.

SEM micrographs confirmed that, using the minimal conditions necessary to achieve a readable QR code, the ablation depth was no more than 50–70 µm. This enabled the inner functional coating layer to remain intact, as it displayed for Opadry^®^ TC Brown (Figure 13a–c) and TF Brown (Figure 13d–f). Furthermore, only physical destruction of the outer layer could be detected with no visible signs of chemical changes, such as burning. This was confirmed by Raman studies (see Section 3.8.7). The API’s release profile is not expected to be negatively affected by the observed physical changes, but we have tested this as well (see Section 3.8.6).

#### 3.8.5. Physical Properties

The physical properties of tablets with increased coating thickness were also evaluated before and after laser treatment. The conditions of laser marking were 280 µJ, 150 pulses; 200 µJ, 140 pulses; and 280 µJ, 90 pulses for Opadry^®^ TC, TF Brown, and TC Blue coatings, respectively.

Table 6 summarizes the results of hardness, friability, and disintegration time of tablets with each coating type. Similar to the previous batches, the hardness decreased, while friability remained constant after laser processing, with no considerable difference between various types of coatings. Nevertheless, in present case, the disintegration properties were not affected by laser treatment, which supports the results of SEM and surface profile measurements and confirms that the increased coating thickness enables the laser marking of functionally coated tablets.

#### 3.8.6. In Vitro Dissolution Test

**Figure 14:**
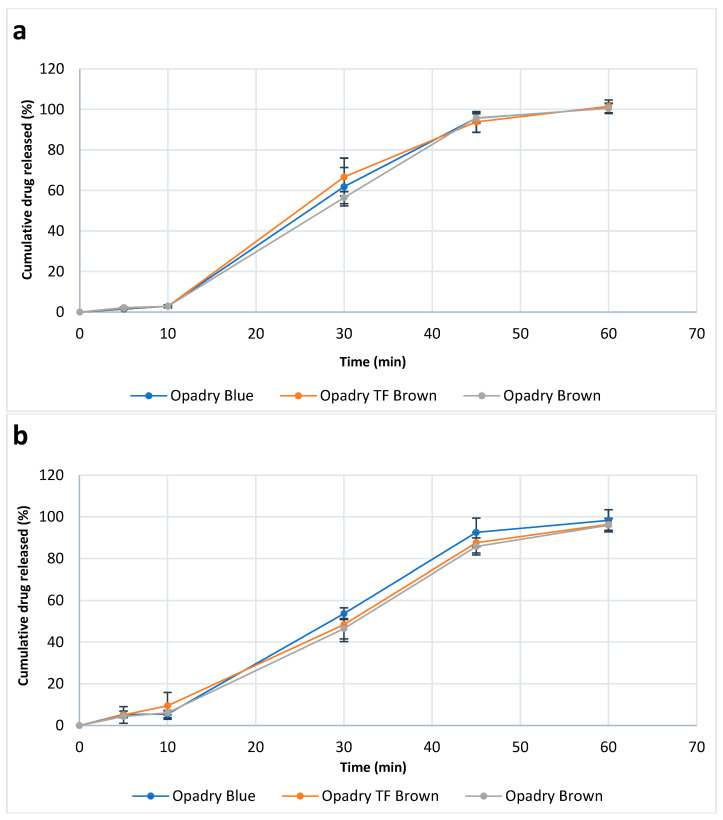
Ibuprofen release profiles of film-coated tablets with increased coating thickness (**a**) before and (**b**) after laser ablation (n = 6).

The same previously lasered tablets were also tested here. Figure 14 displays the release profile of the tablets before and after laser marking. As it was expected based on the results of the physical characterization, all tablets remained intact in the gastric medium for 2 h, without considerable leakage of the API, followed by complete dissolution in the basic medium within 60 min. These results are consistent with the Ph. Eur. requirements for delayed release tablets due to the presence of an enteric coating (i.e., Acryl-EZE^®^ MP). These findings also support SEM observations regarding the structural integrity of the functional coating layer in the same formulation.

In addition, no significant difference was observed between various coatings (*p* ˃ 0.05), so the presence/absence of TiO_2_ and the general effect of laser treatment did not affect the release pattern.

However, this finding was partially in contrast to the findings of several research articles, which have shown how radiation affected the properties of polymers and the ability to control the release of SDFs [99,100].

Machiste et al. demonstrated that the observed changes in release profiles of diltiazem from hydrophilic matrices depended on polymer type and UV laser exposure time. For example, drug release from polyvinyl alcohol (PVA)-based matrices was not affected by UVA radiation. However, significant differences were observed between hypromellose (HPMC) and polyethylene oxide (PEO), as PEO was more affected than HPMC according to rheological analysis. They interpreted the results by scissoring of polymer chains due to laser exposure, thus reducing their molecular weight and the corresponding viscosity [101]. It is known that the high photon energy of UV lasers may be responsible for photochemical reactions, which are supposed to result in breaking the chemical bonds of polymers [37]. The presence of oxygen bridges in the PEO chain makes it susceptible to photocatalytic damage. Although this polymer is transparent to UV-A radiation, the presence of oxygen or ketones as impurities allows for photocatalytic hydrogen removal. For instance, the presence of oxygen leads to the formation of hydroperoxides, which in turn undergo photocatalytic O-O bond cleavage, generating hydroxyl and alkoxyl radicals, known for their chain-degrading properties. In contrast, irradiation under anaerobic conditions is only effective at short wavelengths. In addition, this effect is more pronounced with linear polymer chains, especially when starting with the short ones. Despite the chemical similarity among the previous polymers, the more complex cyclic structure of HPMC is less susceptible to damage per hydrogen removal. Hydrogen removal is preferred at the alpha site of HPMC, where the resulting hydroxyl alkyl radical loses a hydrogen atom, initiating chain degradation. However, PVA is only slightly sensitive to light. Therefore, the presence of a catalyst such as TiO_2_ is necessary for photodegradation to occur [101].

Titapiwatanakun et al. indicated the possibility of improving the control of enteric coating systems by using a carbon dioxide (CO_2_) laser. This could result in better therapeutic outcomes by reducing individual variations among patients and ensuring that the drug is delivered at the right place and time. In this study, laser-ablated tablets showed faster dissolution, and shorter lag times were observed compared to untreated samples. This can be due to the laser-induced changes in physicochemical, morphological, and mechanical properties of the studied films. CO_2_ laser irradiation results in fast melting and localized, precise re-solidification/vaporization of polymers through absorption of IR energy, which can damage the integrity and function of enteric film coatings. These changes result primarily from either the formation of pores on the film surface and/or detachment of the film layer. In addition, it was found that various grades of Eudragit exhibited different behavior after this processing. Eudragit L100-55 was the most affected, and this was explained by the sensitivity of the side chains (i.e., chain length) and the presence of acryl units instead of methacrylate, which are less reactive to laser irradiation [98]. Litauszki et al. successfully controlled drug release from polylactic acid (PLA) films by setting the excimer laser energy. This is important for modulating the drug release from subcutaneous implants or vaginal applications. Direct, non-thermal excitation of PLA chains is expected at 248 nm without significant damage or free radical generation in drug molecules (caffeine). The study results showed that increasing the number of laser pulses led to increased photolysis (splitting of the polymer chain), resulting in a decrease in the molecular weight of this polymer and an increase in the drug release rate. However, this relationship exhibits a characteristic saturation-like behavior. For example, the rate and amount of caffeine released increase up to 2000 laser pulses [102].

#### 3.8.7. Raman Spectra

Raman measurements were performed on the halved surface of the same, previously lasered tablets (280 µJ, 150 pulses; 200 µJ, 140 pulses; and 280 µJ, 90 pulses for Opadry^®^ TC, TF Brown, and TC Blue), exclusively in the core region. For all three formulationS, six measurement points (A1–A6 and B1–B6) were examined before and after laser treatment, as indicated in Figure 3. Since the tablet core contains several components in addition to the API, such as fillers, binders, and lubricants (e.g., talc, microcrystalline cellulose, and magnesium stearate), the spectra obtained at the six points were averaged for comparison.

**Figure 15:**
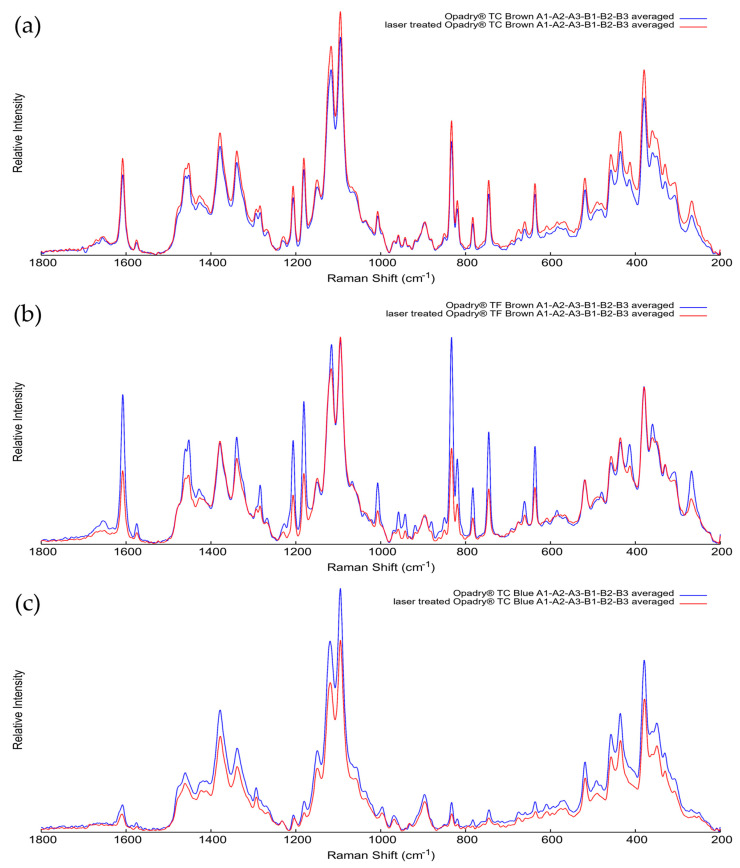
Averaged Raman spectra of the tablet cores before (blue lines) and after (red lines) laser treatment for (**a**) Opadry^®^ TC Brown, (**b**) Opadry^®^ TF Brown, and (**c**) Opadry^®^ TC Blue coated tablets with increased coating thickness.

Figure 15 shows that the averaged spectra of the non-treated and laser-treated tablet samples overlap well with no significant differences in the characteristic band positions or relative intensities. This indicates that the laser treatment did not cause any detectable chemical changes in either ibuprofen or the tablet core matrix. Based on the shape and position of the spectral bands, the laser did not cause any local structural rearrangement attributable to thermal effects.

**Figure 16:**
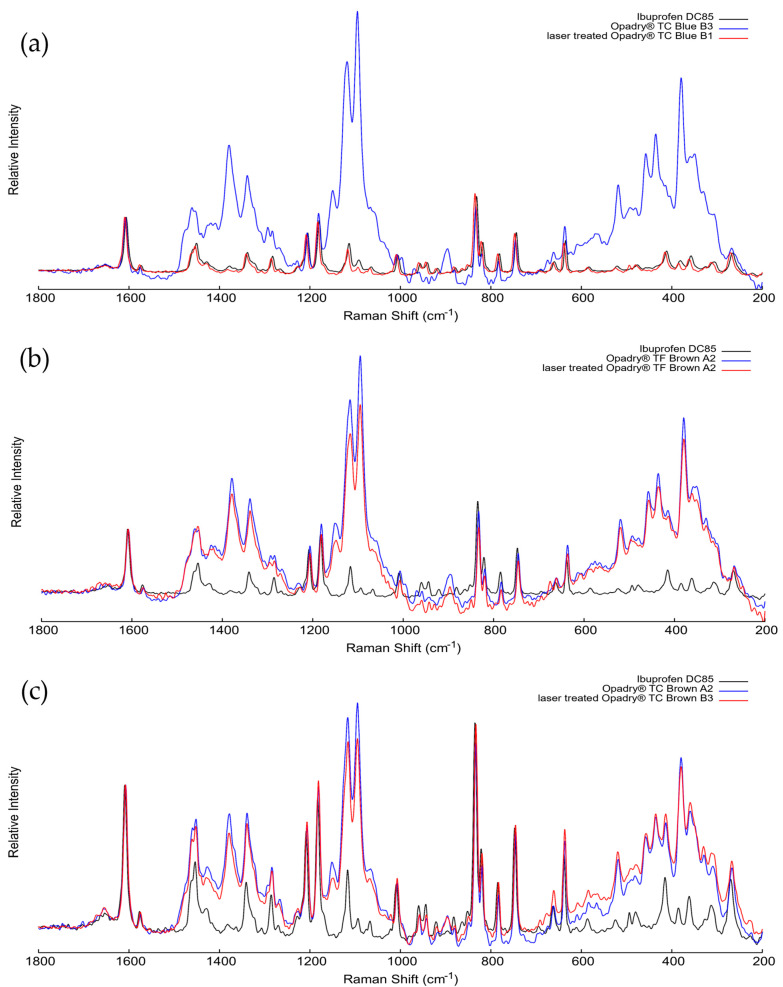
Point-specific Raman spectra recorded directly from ibuprofen crystals within the tablet cores, compared with the pure ibuprofen DC 85 reference (black). (**a**–**c**) Spectra of untreated (blue) and laser-treated (red) tablets with increased coating thickness were normalized to the C=C stretching band at ~1604 cm^−1^.

In addition to the averaged spectra, individual spectra were also collected from the tablet cores to compare them directly with the pure ibuprofen reference, as shown in Figure 16. These measurements were performed at sites where the laser spot coincided with visible ibuprofen crystals within the core, enabling direct detection of the active ingredient’s Raman signal.

For all three formulations, spectra of non-treated tablet, the laser-treated tablet, and pure ibuprofen DC 85 were normalized to the characteristic band at 1604 cm^−1^, which corresponds to the C=C stretching of the aromatic ring of ibuprofen. This diagnostic peak appeared consistently in all samples, independent of laser treatment. The constancy of the peak position and relative intensity confirms that the chemical structure of the active ingredient remained unaffected by laser processing.

After normalization, the spectral profiles of pure, unlasered, and laser-treated samples showed almost complete overlap, indicating that no oxidation, degradation, or polymorphic transformation occurred. These point-specific measurements are therefore consistent with the results of the averaged spectra and confirm that, under the laser parameters used, ibuprofen remained chemically and structurally stable during the laser ablation process.

Last but not least, Table 7 summarizes the key results of the formulations prepared with increased thickness and final optimized laser parameters for each coating.

## 4. Conclusions

The present work demonstrates that ultrafast Ti:Sa femtosecond laser marking can produce readable, traceable QR codes (Version 1, 5 × 5 mm; up to 25 alphanumeric characters at medium error correction) directly on the surface of double film-coated ibuprofen tablets at industrially relevant speeds (1.5–7 s per tablet). By optimizing both the laser parameters (pulse energy: 200–280 µJ; number of pulses: 90–150) and the coating architecture (enteric layer thickness increased to 200–250 µm), we achieved selective ablation of the outer Opadry^®^ layer (50–70 µm depth) while preserving the functional integrity of the inner Acryl-EZE^®^ MP gastro-resistant coating. In vitro disintegration and dissolution tests, SEM imaging, and Raman spectroscopy confirmed that the optimized formulations have maintained gastro-resistance (>120 min in pH 1.22 medium) and complete API release in pH 6.82 buffer, with no detectable chemical degradation of ibuprofen or the polymer matrix.

The results demonstrated that ultrafast Ti:Sa laser marking appears to offer a range of advantages for the design of SDFs. In addition to product recognition, this type of marking may be an effective, fast, and economical strategy for tracking and combating drug counterfeiting in the pharmaceutical industry. The results of this study support the idea that laser marking can also play a critical role in the field of personalized medicines.

These results support the safe implementation of direct-on-dose-form data carriers as a complementary strategy to packaging serialisation with the requirements of the EU Falsified Medicines Directive: EU Directive 2011/62/EU and Commission Delegated Regulation (EU) 2016/16, particularly for small, high-value batches, hospital/clinical personalization, and enhanced patient verification in the absence of original packaging.

However, careful balancing of formulation (coating thickness and pigment type) and laser processing conditions is essential, especially for delayed-release systems.

Further investigation should be conducted when laser marking is applied to film-coated tablets with a single layer, in case of light-sensitive APIs, or with different types of lasers, processing parameters, and formulations. In addition, due to limited laser facility access, a full Design of Experiments (DoE) analysis was not feasible. Parameters were optimized independently for each coating type to achieve readability; future studies will employ response surface methodology to map the design space systematically.

Nevertheless, the results presented herein establish a robust foundation for the safe and efficient implementation of laser-based direct-on-dose QR code marking in pharmaceutical manufacturing and anti-counterfeiting efforts.

### Future Perspectives

Beyond anti-counterfeiting, tablet-level QR coding offers transformative opportunities for pharmaceutical practice. Integration with the Internet of Things (IoT) platforms could enable real-time supply chain transparency, with each tablet’s QR linked to a blockchain-secured database for traceability from manufacture to patient administration. Smart packaging equipped with NFC/RFID readers could automatically log medication intake, supporting adherence monitoring and clinical trials. For personalized medicine, dynamic QR generation in hospital pharmacies—encoding patient-specific dosing, allergy warnings, or drug interaction alerts—would improve medication safety, particularly for pediatric, geriatric, or polypharmacy populations. Tablet-level QR coding can extend beyond anti-counterfeiting to IoT/blockchain traceability and smart-packaging adherence logging; patient-specific codes in hospitals with ePIL links improve safety. Next-generation ultrashort-pulse, multiwavelength lasers plus ML-guided PAT may enable real-time, coating-independent optimization. The solvent-free process supports sustainability; standards and stability validation will require joint industry–regulator action.

## Figures and Tables

**Table 1:** Coating parameters of Acryl-EZE^®^ MP.

Step	Inlet Air Temperature (°C)	Exhaust Air Temperature (°C)	Product Temperature (°C)	Drum Speed (rpm)
Preheating	50	-	Until 40	3
Coating	38–40	32.4	30–32	10
Drying	35	27–30	32	3
Cooling	25	25	25	3

**Table 2:** Coating parameters of Opadry^®^ (TC Blue, TF Blue, TC Brown, and TF Brown).

Step	Inlet Air Temperature (°C)	Exhaust Air Temperature (°C)	Product Temperature (°C)	Drum Speed (rpm)
Preheating	65	-	Until 50	3
Coating	52	45.4	42–45	10
Drying	35–40	30–35	27	3
Cooling	25	25	25	3

**Table 3:** QR code capacity table: maximum alphanumeric characters by version and error correction level.

Version	Matrix Size	Error Correction Level			
		Low (~7%)	Medium (~15%)	Quartile (~25%)	High (~30%)
1	21 × 21	25	20	16	10
2	25 × 25	47	38	29	20
3	29 × 29	77	61	47	35
4	33 × 33	114	90	67	50
5	37 × 37	154	122	87	64

**Table 4:** Physical properties of uncoated and coated ibuprofen tablets before laser processing.

Physical Property	Uncoated Tablets	Coated Tablets				
		Acryl-EZE^®^	Opadry^®^ TF Blue	Opadry^®^ TC Blue	Opadry^®^ TF Brown	Opadry^®^ TC Brown
Moisture content (%)	4.03 ± 0.07	3.98 ± 0.13	3.67 ± 0.32	3.58 ± 0.16	3.66 ± 0.09	3.63 ± 0.31
F * (%)	0.31	0				
H ** (N ***)	89.6 ± 4.57	156.8 ± 6.77	228.4 ± 8.14	221.1 ± 7.72	225.1 ± 6.85	231.3 ± 8.32
DT **** in gastric juice (min)	1.13	Gastro-resistant for 120				
DT in phosphate buffer (min)	-	30.20	25.41	26	24.30	25.52

* F = friability, ** H = hardness, *** N = newton, **** DT = disintegration time.

**Table 5:** The physical properties of the film-coated tablets before and after laser ablation.

Physical Property	Acryl-EZE^®^ MP + Opadry^®^ TC Blue		Acryl-EZE^®^ MP + Opadry^®^ TF Brown		Acryl-EZE^®^ MP + Opadry^®^ TC Brown	
	Before	After	Before	After	Before	After
H (N)	221 ± 7.72	114.7 ± 2.98	225 ± 6.85	117.45 ± 8.95	231 ± 8.32	125.65 ± 7.38
F (%)	0					
DT in gastric juice (min)	Gastro-resistant for 120	0.83	Gastro-resistant for 120	1.15	Gastro-resistant for 120	0.96
DT in phosphate buffer (min)	26	-	24.30	-	25.52	-

**Table 6:** The physical properties of the film-coated tablets with increased coating thickness before and after laser treatment.

Physical Property	Acryl-EZE® MP + Opadry® TC Blue		Acryl-EZE® MP + Opadry® TF Brown		Acryl-EZE® MP + Opadry® TC Brown	
	Before	After	Before	After	Before	After
H (N)	245.2 ± 7.91	201.2 ± 3.96	240.8 ± 4.76	197.2 ± 8.46	249.6 ± 8.64	201.6 ± 9.04
F (%)	0					
DT in gastric juice (min)	Gastro-resistant for 120					
DT in phosphate buffer (min)	40.93 ± 6.98	37.54 ± 9.68	43.33 ± 2.51	40.26 ± 4.52	48.66 ± 1.52	43.03 ± 4.01

**Table 7:** Optimized laser parameters and outcomes for double film-coated tablets with increased coating thickness.

Coating Type	Pulse Energy (µJ)	Number of Pulses	Ablation Depth (µm)	QR Readability	Gastro-Resistance Preserved
Opadry^®^ TC Blue	280	90	50–60	Readable	Yes (>120 min pH 1.22)
Opadry^®^ TF Brown	200	140	50–70	Readable	Yes (>120 min pH 1.22)
Opadry^®^ TC Brown	280	150	50–60	Readable	Yes (>120 min pH 1.22)

## Data Availability

The datasets presented in this article are not readily available because the data are part of an ongoing study and it requires further time until the raw data can be transformed to publicly accessible file formats, the file names and nomenclature used in the various measurements would be unified, and the correct metadata can be provided to enable the correct submission of the raw files into a publicly available data repository. Until that time, requests to access the datasets should be directed to the corresponding author (sovany.tamas@szte.hu).

## Supplementary Materials

The following supporting information can be downloaded at: https://www.mdpi.com/article/10.3390/pharmaceutics18010073/s1, Table S1: Experimental laser parameters applied to the film-coated tablets with readability results. Table S2: Experimental laser parameters applied to tablets with increased coating thickness and their readability results.
