# Supplementary material for: Laser-Based QR Code Marking on Double Film-Coated Tablets: Balancing Marking Efficiency and Tablet Integrity—A Step Toward Safer Medicines
Source: Pharmaceutics. 2026 Jan 6;18(1):73. doi: 10.3390/pharmaceutics18010073 (PMC12845212; doi:10.3390/pharmaceutics18010073)
Supplement: Supplementary file 1 [file pharmaceutics-18-00073-s001.zip › pharmaceutics-4043596-supplementary.pdf]

Table S1. Experimental laser parameters applied to the film-coated tablets with readability results.

Laser parameters		Opadry® coating type			
Energy (μJ)	Number of pulses	TF Blue	TC Blue	TF Brown	TC Brown
400	30		0*		
	50		1**		
	80		1		
375	30		0		
	50		0		
355	50		0		
	60		1		
350	20			0	
	30			1	1
	40			1	1
	50	0	1	1	1
	60	0	1	1	
	70	0		1	
	75			1	
300	25			0	
	30		0	1	1
	50		0		
	60		1		
	70		1		
	75		1		
	80	0	1		
	100	0			
290	60		0		
	70		0		
	80		1		
280	30			1	
	35			1	
	75		1		
	80		1		
265	20				1
	25			0	
	30			1	1
	35			1	
	80		1		
	85		1		
250	55		0		
	65		0		
	75		0		
245	20				%
	25			0	%
	30			%***	1
	35			1	1
	80		1		
	85		1		

	90	1
225	65	0
	70	0
	75	0

*0 = unreadable, **1 = readable, ***% = partially readable.

Table S2. Experimental laser parameters applied to tablets with increased coating thickness and their readability results.

Laser parameters		Opadry® coating type		
Energy (μJ)	Number of pulses	TC Blue	TF Brown	TC Brown
280	75		1	
	80	0*		
	90	1**		
	100	1		%
	150	1		1
250	150	%***	%	%
245	80	0		
	100		1	
	120		1	
	150	%		%
200	100	0	%	0
	120	0	%	0
	140	0	1	0
	160	0		0
	180	0		0
	200	0		0
	220	0		0
	240	0		0

*0 = unreadable, **1 = readable, ***% = partially readable
